# Dietary Olive Oil Intake Improves Running Endurance with Intramuscular Triacylglycerol Accumulation in Mice

**DOI:** 10.3390/nu13041164

**Published:** 2021-04-01

**Authors:** Yusuke Komiya, Makoto Sugiyama, Masaru Ochiai, Nanako Osawa, Yuto Adachi, Shugo Iseki, Keizo Arihara

**Affiliations:** 1 Department of Animal Science, School of Veterinary Medicine, Kitasato University, Towada 034-8628, Japan; mochiai@vmas.kitasato-u.ac.jp (M.O.); ag107093@gmail.com (N.O.); va16002@st.kitasato-u.ac.jp (Y.A.); va17012@st.kitasato-u.ac.jp (S.I.); arihara@vmas.kitasato-u.ac.jp (K.A.); 2 Faculty of Veterinary Medicine, School of Veterinary Medicine, Kitasato University, Towada 034-8628, Japan; masugi@vmas.kitasato-u.ac.jp

**Keywords:** olive oil, skeletal muscle, IMTG, DGAT1

## Abstract

Olive oil is a functional food shown to have a variety of bioactive effects. Therefore, we expect it to be a novel functional food with an exercise-mimetic effect on skeletal muscles. This study aimed to investigate the effect of olive oil on the endurance capacity and muscle metabolism in mice. Mice fed a 7% (*w*/*w*) olive oil diet for eight weeks showed improved treadmill running endurance and increased intramuscular triacylglycerol (IMTG) accumulation in the gastrocnemius muscle compared to soybean oil diet-fed controls. The increase in running endurance with olive oil intake was independent of the muscle fiber type. To elucidate underlying the mechanism of elevated IMTG levels, we examined the expression levels of the genes related to lipid metabolism. We found that the expression of diacylglycerol O-acyltransferase1 (DGAT1) was significantly upregulated in the muscle of olive oil diet-fed mice. In addition, the olive oil diet-fed mice showed no metabolic impairment or differences in growth profiles compared to the controls. These results suggest that dietary olive oil intake affects muscle metabolism and muscle endurance by increasing energy accumulation.

## 1. Introduction

Two major muscle fiber types exist in mammalian skeletal muscle: type 1 (slow-twitch oxidative, red muscle) and type 2 fibers (fast-twitch glycolytic, white muscle) [1]. Type 1 fibers contain more mitochondria, possess a high oxidative capacity, and are resistant to fatigue whereas type 2 muscle fibers show high rates of glycolytic metabolism and fatigue. Thus, muscles enriched in type 1 fibers typically perform sustained and tonic contractile activities, such as postural tension, whereas muscles enriched in type 2 fibers are typically involved in intense and rapid activities of short duration. In other words, the composition of muscle fiber types determines a variety of skeletal muscle properties, including muscle endurance (fatigue-resistant or fatigable), contraction (slow-twitch or fast-twitch), and metabolic characteristics (oxidative or glycolytic).

Intramuscular triacylglycerol (IMTG), including intramyocellular lipids (IMCL), which are lipids stored in skeletal muscle tissue, is abundant in oxidative muscle fibers and is a key factor to understand muscle endurance capacity [2]. Furthermore, it has been shown that the IMTG pools adaptively increase in response to exercising and are higher in endurance-trained individuals, which could be because, during endurance exercise, the increased demand of muscles for adenosine triphosphate (ATP) is met through continuous regeneration of ATP in mitochondria through the oxidation of fatty acids derived from IMTG pools [3,4,5,6]. However, recent studies have reported that food ingredients that are functional for muscle properties could mimic the effects of exercise.

Dietary oils rich in monounsaturated fatty acids (MUFA), which are the healthy fatty acids, are widely used in cooking, because of the health consciousness and improved lifestyle of people. Therefore, studies on dietary fats have garnered a lot of interest in nutrition research. Olive oil, rich in MUFAs, is the main cooking oil in the Mediterranean countries, and its consumption, specifically the extra virgin variety, is associated with several biological activities, including anti-dyslipidemia, anti-obesity, anti-diabetic, antioxidant, anti-atherogenic, anti-hypertensive, anti-inflammatory, and hepatoprotective actions [7]. The impact of olive oil intake on skeletal muscles remains unknown, even though several biological effects have been identified. To the best of our knowledge, a single study has reported that olive oil intake upregulates uncoupling protein genes in rat skeletal muscles [8]. This report indicates that olive oil intake, albeit under high-fat conditions, may induce changes in muscle metabolism. Furthermore, we have reported that dietary fish oil intake mimics the effect of exercise, i.e., a transition from type 2B to type 2X fibers and an increase in oxidative metabolism factors in the extensor digitorum longus (EDL) muscles of rats [9]. 

Considering these facts, we hypothesized that daily consumption of foods enriched in certain dietary fats, such as olive oil, could also provide an exercise-inducing effect. To test this hypothesis, the present study was aimed to investigate the effects of dietary supplementation of olive oil on muscle metabolism and endurance capacity.

## 2. Materials and Methods

### 2.1. Animals and Diets

Eight-week-old male C57BL/6JJcl mice were purchased from CLEA Japan Inc. (Tokyo, Japan). Mice were housed in plastic cages in an animal room at 22 ± 2 °C and 50 ± 10% humidity under an artificial lighting system of 12-h light and 12-h darkness (lights on from 08:00 to 20:00). The mice were acclimated to the environment for a week. Following the acclimatization period, the mice were fed each diet based on the AIN-93G composition containing 7% (*w*/*w*) soybean oil (control diet) or fully refined olive oil (free from polyphenols) for 8 weeks. Diets were purchased from Research Diet, Inc. (New Brunswick, NJ, USA). Detailed components of diet and fatty acid composition are shown in Table 1 and Table 2, respectively. All animal experiments were conducted in strict accordance with the recommendations in the Guidelines for Proper Conduct of Animal Experiments published by the Science Council of Japan, and with the approval of the Animal Care and Use Committee of Kitasato University (approval no. 16-162 and 19-098).

### 2.2. Experimental Procedures

The mice were housed with each diet for 8 weeks. Between the 6th and 7th weeks, the running endurance test, oral glucose tolerance test (OGTT), and insulin tolerance test (ITT) were performed. After an 8 week feeding period, the mice were sacrificed. Blood was collected and centrifuged to obtain serum. Adipose tissues, liver, and skeletal muscles (gastrocnemius, soleus, and EDL muscles) were harvested immediately and weighed. The gastrocnemius muscles were frozen and crushed in liquid nitrogen. Serum and all tissues were stored at −80 °C until analysis.

### 2.3. Treadmill Endurance Test

Mice were acclimated to treadmill running (10 m/min for 10 min) two days before the test. For the endurance test, the mice were run on a treadmill at a 5° incline, and the speed was gradually increased from 10 m/min to 15 m/min. After reaching 15 m/min, the mice were exhausted at a constant speed. Endurance was measured as a function of time and distance.

### 2.4. Serum Biochemical Analysis

#### 2.4.1. Glucose, Triacylglycerol (TAG), and Non-Esterified Fatty Acid (NEFA) Analyses

The levels of serum glucose, TAG, and NEFA were measured using commercial kits (LabAssay^TM^ Glucose: 298-65701, LabAssay^TM^ Triglyceride: 290-63701, LabAssay^TM^ NEFA: 294-63601, FUJIFILM Wako Pure Chemical Corporation, Osaka, Japan).

#### 2.4.2. Oral Glucose Tolerance Test (OGTT) and Insulin Tolerance Test (ITT)

OGTT: After overnight fasting, d-glucose (1 g/kg) was orally administered to each mouse. Blood was collected from the tail vein before and after 15, 30, 60, 90, and 120 min of d-glucose administration and centrifuged to obtain plasma to measure glucose levels.

ITT: After overnight fasting, porcine insulin (0.33 IU/kg) was intraperitoneally administered to each mouse. Blood was collected, and plasma was obtained before and after 15, 30, 60, 90, and 120 min of insulin administration.

The plasma glucose levels during the OGTT and ITT were measured using a commercial kit (Fujifilm Wako Pure Chemicals Ltd., Osaka, Japan). The area under or over the curve (AUC or AOC) of the plasma glucose levels during the OGTT and ITT were calculated using the trapezoidal rule.

### 2.5. Muscle TAG Content

#### 2.5.1. Biochemical Determination

Total lipids were extracted using the Folch method [10], with minor modifications. Crushed gastrocnemius muscle tissues (approximately 50 mg) were homogenized in 1.5 mL of chloroform/methanol (2/1, *v*/*v*), and incubated overnight at 4 °C. Then, 250 μL of 0.9% NaCl solution was added to the incubated chloroform/methanol solutions and mixed by vortexing. After centrifugation, 600 μL of the lower phase containing neutral lipids was dried using a depression aspirator (A-3S; Tokyo Rikakikai Co., Ltd., Tokyo, Japan). To determine the TAG content, the lipid pellet was solubilized in Triton X-100, and TAG concentrations were determined using a commercial kit (Fujifilm Wako Pure Chemicals Ltd.) following the manufacturer’s instructions. Muscle TAG content was normalized to wet muscle tissue weight.

#### 2.5.2. Preparation of Muscle Sections and BODIPY 493/503 Staining

The EDL and soleus muscles were fixed overnight at 4 °C with 4% paraformaldehyde. After fixing, samples were equilibrated in 10%, 20%, and 30% sucrose in phosphate-buffered saline (PBS) at each concentration for 2–3 h at 4 °C and embedded in Tissue-Tek OCT Compound (Sakura Finetek Japan, Tokyo, Japan). The embedded samples were frozen in isopentane and cooled with liquid nitrogen. Cryosections from each block were collected on Matsunami adhesive silane (MAS)-coated glass slides (S9226; Matsunami Glass Ind., Ltd., Osaka, Japan). IMTG were stained with BODIPY 493/503 (Life Technologies, Carlsbad, CA, USA), a dye for neutral lipids, and a tracer for oils and other nonpolar lipids, according to described methods [11] with minor modifications. Briefly, BODIPY 493/503 was diluted in 10% (*v*/*v*) dimethyl sulfoxide in PBS at a concentration of 1 mg/mL. Muscle specimens were immersed in BODIPY 493/503 solution for 90 min. The samples were then washed three times with PBS for 10 min. All samples were mounted in ProLong^TM^ Gold antifade reagent with DAPI (Invitrogen, Grand Island, NY, USA). Stained IMTG was visualized using a confocal microscope LSM 710 (Carl Zeiss, Oberkochen, Germany). Calculations of IMTG content were performed using ImageJ 1.45 f software (Rasband W; National Institutes of Health, Bethesda, MD, USA) by the densitometry of BODIPY 493/503 fluorescence intensity and standardized to the area.

### 2.6. RNA Isolation and Reverse Transcription-Quantitative Polymerase Chain Reaction (RT-qPCR)

Total RNA was extracted from crushed gastrocnemius muscle tissues using the ISOGENII kit (NIPPON GENE, Tokyo, Japan), and reverse transcribed using SuperScript III Reverse Transcriptase (Invitrogen) and Oligo d(T)12–18 primers (Applied Biosystems, Waltham, MA, USA) according to the manufacturer’s instructions. The following genes and transcription factors were selected: transcriptional factors (SREBP1, PPARγ, PPARδ, and PPARα), TAG synthesis (ACSL1, DGAT1, and DGAT2), FA import (CD36), de novo FA synthesis (FASN and ACACA), lipid droplet formation (PLIN5), lipolysis (hormone-sensitive lipase (HSL), LPL, and ATGL), FA metabolism (CPT1β and PDK4), glycometabolism (GCK, GLUT4, and MPC1), and mitochondria (UCP3 and PGC1α). RT-qPCR was performed using the Applied Biosystems StepOne Plus system using the PowerUp SYBR Green Master Mix (Thermo Fisher Scientific, Waltham, MA, USA). All primers were designed using the ProbeFinder software (version 2.53; Roche Diagnostics GmbH, Mannheim, Germany) with an intron-spanning assay. The primer sets used in this study are listed in Table 3. Amplicon specificity was verified using a melting curve analysis. TATA-box-binding protein (TBP) was used as an internal standard.

### 2.7. Western Blotting

Crashed gastrocnemius muscles (approximately 50 mg) were homogenized in SDS solution containing 10% SDS, 40 mM DTT, 5 mM EDTA, and 0.1 M Tris-HCl buffer (pH 8.0), in which a protease inhibitor cocktail for use with mammalian cell and tissue extracts (Nacalai Tesque, Inc., Kyoto, Japan) was added at 1:100. The sample homogenates were heated in boiling water for 3 min. Total protein concentrations were assayed using Pierce BCA Protein Assay Reagent (Thermo Fisher Scientific, Waltham, MA, USA). The final protein concentration was 8 μg/μL. The prepared protein samples were separated via 10% sodium dodecyl sulfate-polyacrylamide gel electrophoresis (SDS-PAGE) under reducing conditions and transferred onto PVDF membranes (Bio-Rad, Hercules, CA, USA). The membranes were then incubated with a blocking reagent (5% powdered skim milk in TTBS) for 45 min before incubation with primary antibodies diluted in CanGetSignal solution 1 (Toyobo, Osaka, Japan) overnight at 4 °C. The following antibodies were used: rabbit monoclonal anti-β- actin (HRP conjugate) (Cell Signaling Technology, #5125 (13E5), 1:1000), rabbit polyclonal anti-DGAT1 (Novusbio, NB110-41487, 1:500), anti-PDK4 (Proteintech, 12949-1-AP, 1:2000), anti-SREBP1 (Abcam, ab28481, 1:1000), and anti-UCP3 (Abcam, ab3477, 1:2000). The membranes were then incubated for 1 h with a peroxidase-conjugated anti-rabbit IgG secondary antibody (1:5000 dilution; Dako, Santa Clara, CA, USA) diluted in CanGetSignal solution 2 (Toyobo Co., Osaka, Japan). The bands were detected using enhanced chemiluminescence (ECL; GE Healthcare, Chicago, IL, USA). The intensity of the bands was quantified using ImageJ software and normalized to β-actin as a loading control.

### 2.8. Myosin Heavy Chain (MyHC) Isoform Composition

Determination of the MyHC isoform compositions, a molecular marker of muscle fiber type, in gastrocnemius muscles was performed by high-resolution SDS-PAGE as described [12]. Briefly, the protein extracted from the gastrocnemius muscle was prepared at a final concentration of 10 ng/μL in a 1× sample buffer. Acrylamide (acrylamide/bisacrylamide ratio of 99:1) gel (8%) containing 35% glycerol (*v*/*v*) was used as the separating gel. After loading the samples, electrophoresis was performed at a constant voltage of 140 V for 22 h at 4 °C. The gels were stained with Silver Stain Kanto III (Kanto Chemical Co., Inc., Tokyo, Japan). The relative MyHC isoform content was quantified by densitometry using the ImageJ software. MyHC isoforms were identified according to their different migration rates (MyHC1 > 2 B > 2X + 2A). A mixed sample of rat soleus and EDL muscles was used as a reference for the four MyHC isoforms.

### 2.9. Statistical Analysis

Data are represented as mean ± SE. Statistical significance was determined using a two-tailed unpaired Student’s *t*-test with a significance of 0.05 or log-rank Mantel-Cox test (for running population). All statistical analyses were performed using the software Excel-Toukei (version.7.0; Social Survey Research Information Co., Ltd., Tokyo, Japan).

## 3. Results

### 3.1. Olive Oil Intake Improves Treadmill Endurance Capacity in Mice

**Figure 1:**
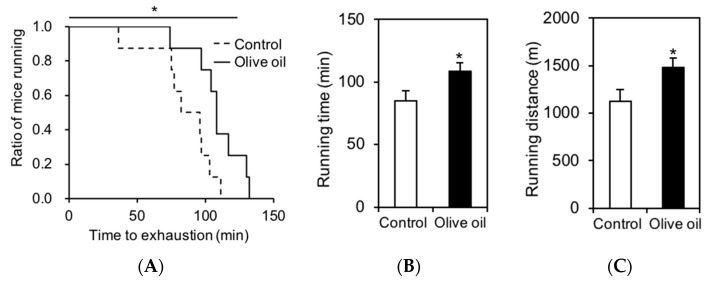
Treadmill running endurance test. (**A**) Running population: ratio of running mice and time. Each data point represents an individual mouse plotted against the time covered by the said mouse at exhaustion. (**B**) Time and (**C**) distance covered until attaining exhaustion under a forced running exercise-to-exhaustion. Results are presented as means ± SE (*n* = 8), *, *p* < 0.05 vs. control group.

We first examined the effects of olive oil diet on mouse activity. After 6 weeks of olive oil feeding and treadmill testing, we found a significant increase in distance running in the mice (Figure 1A). In terms of running time and distance, the olive oil group ran longer (~29%) and further (~32%) than the control group (Figure 1B,C), revealing that dietary olive oil intake increased endurance capacity systemically.

We also examined the effects of olive oil feeding on the physical and serum biochemical parameter in mice. After the treadmill test, olive oil feeding for 8 weeks resulted in no changes in all parameters, including the final body weight, body weight gain, total food intake, food efficiency, and tissue weight (skeletal muscles, adipose tissue, and liver) compared to the control group (Table 4). There were also no significant differences in serum glucose, TAG, and NEFA levels between the two groups. This result indicates that the olive oil-containing diet has the same life-support properties as the normal diet.

### 3.2. Olive Oil Intake Increases IMTG Content in Gastrocnemius Muscle

**Figure 2:**
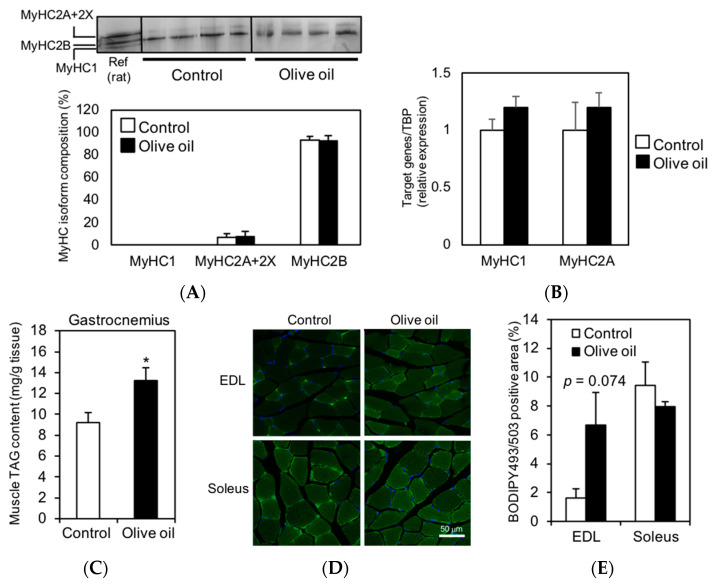
Muscle fiber type compositions and IMTG accumulation in skeletal muscles. (**A**) Separation of myosin heavy chain (MyHC) isoforms by SDS-PAGE analysis. A mixed sample of rat soleus and EDL muscle was used as the three MyHC isoform references (the migration rate was MyHC1 > 2B > 2A+2X). The graph shows the composition of MyHC isoforms in control and olive oil-fed mice. (**B**) Relative expression levels of MyHC1, 2A normalized to TBP expression. (**C**) Whole TAG content in gastrocnemius muscle (mg/g tissue). Results are presented as means ± SE (*n* = 8), *, *p* < 0.05 vs. control group. (**D**) Representative images of muscle specimens stained with BODIPY493/503 in mouse EDL (left panels) or soleus (right panels) muscle. (**E**) Results were calculated BODIPY493/503 positive area/total area. Results presented as means ± SE (*n* = 4).

It has been shown that slow-twitch muscles are superior to oxidative metabolism and endurance capacity [1]. Therefore, we analyzed the MyHC isoform compositions, a fiber-type marker, to determine whether the improvement in running endurance in olive oil-fed mice was due to an increase in slow-twitch muscle; however, there was no significant difference between the two groups (Figure 2A,B). These results suggest that the increase in running endurance with olive oil intake is independent of the muscle fiber type.

Because the IMTG pools have been shown to increase adaptively in response to exercising [5,6], we hypothesized that the increase in IMTG content from olive oil intake would improve endurance capacity. Additionally, in our previous study, we have shown a quantitative correlation between the IMTG and IMCL content [11]. To investigate this hypothesis, we analyzed the IMTG content in the gastrocnemius and the IMCL content in the EDL and soleus muscles. Because we reported that the potential of IMTG accumulation depends on muscle fiber type [11] and these muscles had different muscle fiber types (main expression: gastrocnemius, type 2B; EDL, 2B or 2X; soleus, 2A or 1), they were selected.

TAG accumulation in the gastrocnemius muscle was significantly higher in olive oil-fed mice than in control mice (Figure 2C). Furthermore, quantified IMCL in the EDL and soleus muscles using BODIPY 493/503 staining showed that IMCL was higher in the EDL muscle of the olive oil group than in the control group (*p* = 0.074). However, there was no significant difference in both EDL and soleus muscles between the two groups (Figure 2D,E). These results suggest that olive oil intake increases IMTG content, but the sensitivity of IMTG accumulation depends on skeletal muscle types.

### 3.3. Olive Oil Intake Induces No Metabolic Impairment in Glucose and Insulin Tolerance

**Figure 3:**
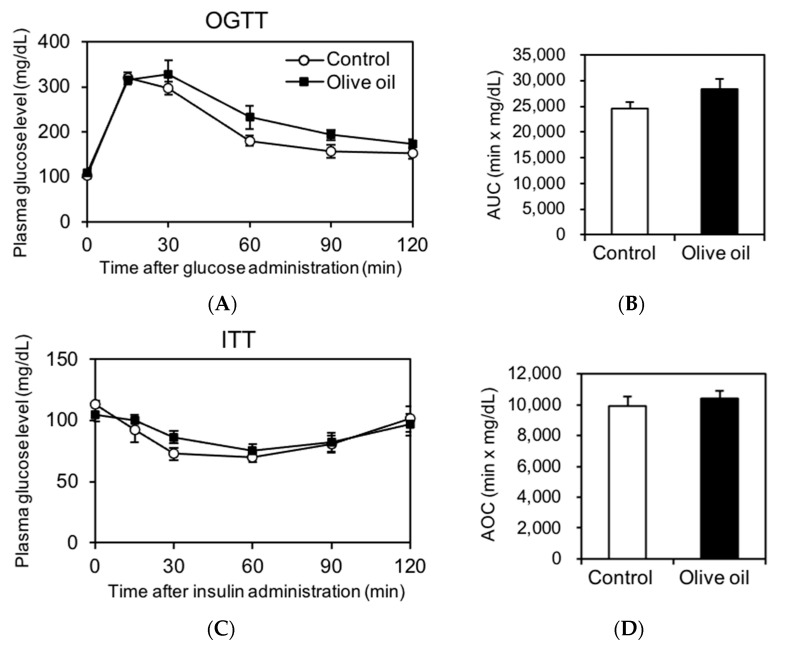
Plasma glucose levels and AUC or AOC values during the OGTT and ITT. Plasma glucose levels during the OGTT (**A**) and ITT (**C**). The area under the curve (AUC: **B**) and area over the curve (AOC: **D**) were determined by the trapezoidal rule. All results are presented as means ± SE (*n* = 8).

Elevated IMTG levels are strongly associated with insulin resistance [5,13]. To elucidate the influence of IMTG accumulation by olive oil intake on insulin resistance, OGTT and ITT were performed. Plasma glucose levels and their AUC and AOC values during the OGTT and ITT are shown in Figure 3. The plasma glucose level, AUC value, and AOC value during both OGTT and ITT did not differ significantly between the two groups (Figure 3A–D). These results suggest that increased IMTG accumulation due to dietary olive oil intake might not impair insulin sensitivity.

Enhanced accumulation of IMTG in muscles has been shown to increase IMTG products such as DAGs, which directly affects the insulin signaling pathway [13,14]. Thus, we attempted to separate and quantify DAGs from lipid extraction in gastrocnemius muscles using high-performance thin-layer chromatography (HPTLC). Separation of DAGs and TAG was observed using iodine staining; however, DAGs were below the limit of detection of the commercial kits used here. Because OGTT and ITT were not significantly different between the two groups (AUC of OGTT: *p* = 0.17, AOC of ITT: *p* = 0.54), we expect the increase in IMTG accumulation is due to the increase in TAG, not DAGs.

### 3.4. Olive Oil Intake Upregulates DGAT1 Expression Levels in Skeletal Muscle

**Figure 4:**
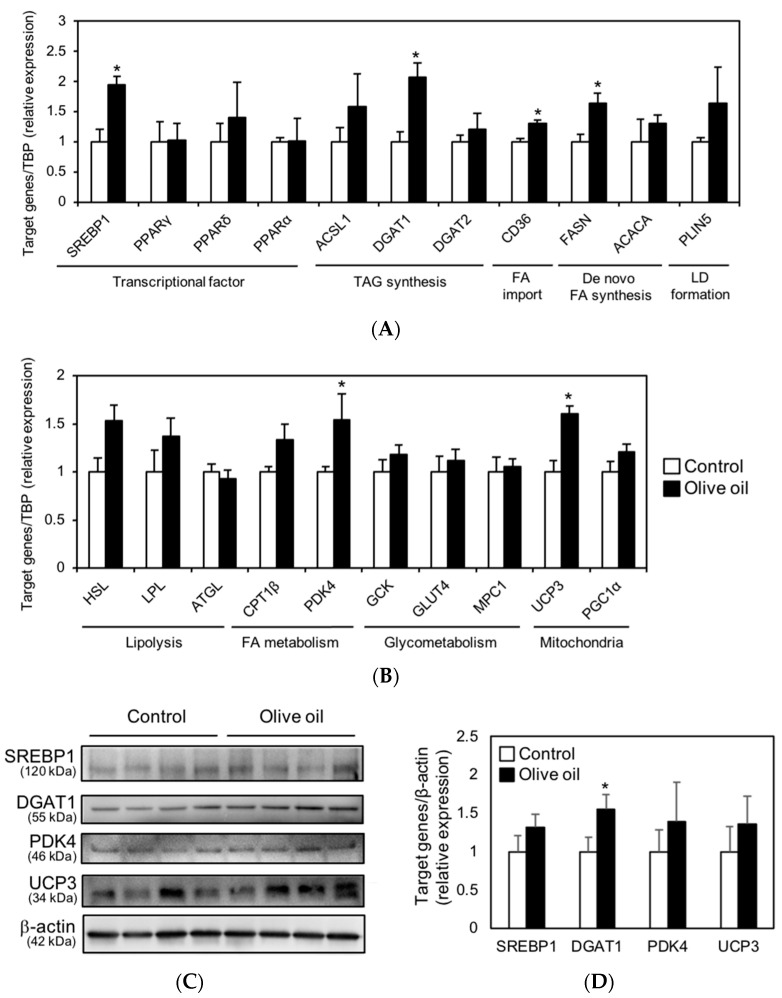
Transcript and protein expression levels in gastrocnemius muscle. (**A**,**B**) Relative expression levels of transcriptional factors (SREBP1, PPARγ, δ, α), genes involved in TAG synthesis (ACSL1, DGAT1, 2), FA import (CD36), de novo FA synthesis (FASN, ACACA), lipid droplet (LD) formation (PLIN5), lipolysis (HSL, LPL, ATGL), FA metabolism (CPT1β, PDK4), glycometabolism (GCK, GLUT4, MPC1), and mitochondrial redox reaction (UCP3, PGC1α). Each gene was normalized to TBP expression. (**C**) Protein expression levels of SREBP1, DGAT1, PDK4, UCP3, and β-actin (loading control). (**D**) The densitometry quantification of panel A. Results are presented as means ± SE (*n* = 8), *, *p* < 0.05 vs. control group.

To understand the muscle TAG accumulation with olive oil intake, we analyzed the transcript levels of genes related to lipid metabolism using RT-qPCR. The transcript expression levels in the gastrocnemius muscle are shown in Figure 4A,B. The transcript levels of SREBP1, DGAT1, CD36, FASN, PDK4, and UCP3 were significantly upregulated in olive oil-fed mice. The upregulated genes are either involved in TAG synthesis (SREBP1 and DGAT1), FA import (CD36), de novo FA synthesis (FASN), FA utilization (PDK4), and heat production (UCP3) in the skeletal muscle. Therefore, we hypothesized that the increase in these factors could have induced both IMTG accumulation and utilization in the olive oil group.

To further reveal the effect of dietary olive oil intake on muscle lipid metabolism, the protein expression of SREBP1, DGAT1, PDK4, and UCP3 in the gastrocnemius muscle was examined. The protein level of DGAT1 was significantly increased in the olive oil group (Figure 4C,D), which corresponded to an increase in transcript levels. However, the protein levels of SREBP1, PDK4, and UCP3 did not increase significantly, contrary to our expectations (Figure 4C,D). 

## 4. Discussion

**Figure 5:**
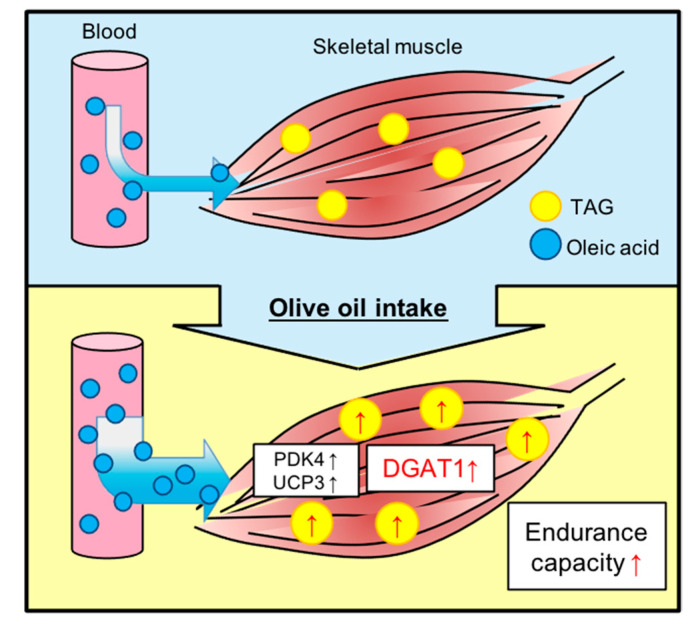
Olive oil improves endurance capacity with increased IMTG pools via upregulation of DGAT1. The upward arrow (↑): upregulation of factors, increase of IMTG content, or improvement of endurance capacity.

The central finding of this study was that dietary olive oil intake in mice resulted in a significant improvement in endurance capacity. We revealed these improvements in endurance were mediated by increased IMTG accumulation in the gastrocnemius muscle. We further showed that IMTG accumulation was induced by upregulation of DGAT1 expression without insulin resistance, as summarized in Figure 5.

The murine gastrocnemius muscle mainly expresses type 2B fibers, while the EDL and soleus muscles express type 2B and 2X, and 2A and type 1 fibers, respectively [11,15]. As the IMTG accumulation potential is superior in the order of type 2A, 1, 2X, and 2B fibers [11], we hypothesized that IMTG accumulation would be found in the soleus muscle (rich in type 2A and type 1 fibers). However, an increase in IMTG content was observed only in the gastrocnemius muscle. In our previous report, we induced IMTG accumulation with a high-fat diet and sciatic nerve denervation [11]. The results demonstrated that IMTG accumulation, induced by muscle inactivity and obesity, leads to metabolic impairment [16,17]. In contrast, in this study, olive oil intake promoted both IMTG accumulation and oxidative metabolism, suggesting that it changes the muscle metabolic property, allowing more lipid utilization, although there was no fiber type change. These metabolic changes in the muscle could induce adaptations that remarkably increase IMTG accumulation in the gastrocnemius muscle, which rarely uses lipids. Notably, the elevated levels of IMTG accumulation induced by high-fat diet intake and sciatic nerve denervation treatment was independent of muscle fiber type composition [11]. Although this mechanism is unclear, the results of this study suggest that there would be an irregular IMTG accumulation mechanism across the composition of muscle fiber type.

In general, an increase in oxidative fibers results in the improvement of muscle endurance. Our results suggested that the increase in running endurance after olive oil intake was independent of the muscle fiber type. There are several reports about the improvement of endurance capacity without a change in the muscle fiber type. For example, in a study on dietary food components, Murase et al. reported that green tea extract-supplemented mice had increased swimming endurance [18]. Interestingly, it has been shown that PPARδ agonism induces a shift in muscle energy substrate utilization (increase in FA metabolism) and increases running endurance by preserving blood glucose levels during exercise [19]. However, the study analyzed the blood glucose levels and respiratory exchange ratio (RER), but not investigate the IMTG levels. Taken together, we hypothesized that this preservation of glucose was due to an increase in the IMTG levels because RER was decreased by PPARδ agonism.

Although olive oil intake increased both IMTG levels and running endurance, it has not been determined whether pre-exercise IMTG content is directly associated with endurance capacity. The skeletal muscle is continuously supplied with energy during exercise from three main substrates: creatine phosphate, glycogen, and TAG. Creatine phosphate is the primary fuel used for short-duration activities, and the anaerobic glycolytic pathway uses muscle glycogen and glucose, which are rapidly metabolized. The oxidative pathway fuels events lasting longer than 2–3 min, and IMTG is mainly used here. Moreover, only the aerobic pathway can produce a large amount of ATP over time via the Krebs cycle and the electron transport system [20]. In addition, lipid oxidation during endurance exercise increases as time passes against decreased carbohydrate oxidation [21]. Because IMTG pools play a key role as an energy substrate in endurance performance, we concluded that increased IMTG pools contributed to improving running endurance in olive oil diet-fed mice. Furthermore, it has been reported that the increased IMTG with physical activity does not show metabolic impairment; however, the elevated IMTG levels are strongly associated with insulin resistance [22]. This paradox has created confusion surrounding the role of the IMTG in muscle metabolism. Therefore, further in-depth investigations are required to understand the precise role of increased IMTG by analyzing the factors related to muscle metabolism besides IMTG accumulation.

SREBP1 is a master regulator of lipid metabolism in skeletal muscle and is present in two forms in vivo: a precursor form (120 kDa) and a mature form (68 kDa) [23]. In the present study, we detected the SREBP1 precursor form, but no significant differences at the protein level were observed with olive oil intake. We also analyzed the expression of the SREBP1 mature form; however, it was not detected. These results suggest that SREBP1 is not a major contributing factor to the increase in the levels of IMTG in the olive oil group. DGAT1 is an enzyme that catalyzes the final conversion of DAG into TAG and plays a vital role in the accumulation of IMTG in the context of exercise [24]. Moreover, it has been shown that the muscle-specific overexpression of DGAT1 leads to the parallel increase in the synthesis of TAG, as well as in fatty acid oxidation (via the upregulation of PDK4 and UCP3), and protects against muscle lipotoxicity [25,26,27]. Therefore, we suggest that the increase in DGAT1 could have induced the accumulation of IMTG and the improvement of the running endurance without metabolic impairment in olive oil-fed mice. Taken together, the results indicate that the olive oil intake mimicked part of the exercise-induced effect via the DGAT1 cascade, explaining why the increase in IMTG did not lead to insulin resistance. 

Factors regulating muscle metabolism are generally activated by mechanical stress and exercise, and it has been shows that dietary ingredients mimic exercising effects by stimulating these factors. For example, amino acids, the representative food components, have been shown to provide exercising effects to muscles—leucine activates the mTORC1 pathway via inhibition of sestrin proteins and promotes muscle hypertrophy through enhanced protein synthesis [28]. Furthermore, dietary components in olive oil have been reported to be functional [7]. For example, oleuropein, a phenolic constituent of olive oil, promotes translocation of GLUT4 into the cell membrane via activation of AMPK and MAPK [29] and protects against H_2_O_2_-induced ROS production in C2C12 myocytes [30]. In addition, we have previously reported that oleic acid, which is abundant in olive oil, improves mitochondrial maximal respiration and increases the transcript expression of oxidative metabolism factors via PPARδ activation [31]. It has also been shown that activation of PPARδ induces a shift in muscle energy substrate utilization (glucose to lipid) and improves endurance capacity by preserving blood glucose levels during exercise [19]. As the olive oil used in this study did not contain oleuropein, it can be inferred that oleic acid could have activated PPARδ, consequently increasing the IMTG levels and endurance capacity. It is noteworthy that the effects of dietary supplementation of olive oil on physical function are due to the combined behavior of multiple fatty acid components present in the oil. Therefore, it is important to identify the specific components to elucidate the underlying active principle, which could find application to design functional foods.

Many studies elucidating the functionality of olive oil and its polyphenols have been conducted under metabolic impairment or high-fat diet conditions [32,33]. We used healthy mice, designed the fed diet using olive oil free from polyphenols to provide standardized calories (not high-fat), and compared the effects using a normal diet to assess the major difference between the two diets in this study was fatty acid composition, and this difference caused the change in muscle metabolism and endurance. Our study approach will contribute to the elucidation of the effects of different diets on physical functions in daily life.

## 5. Conclusions

In the present study, we revealed that dietary olive oil intake improves running endurance with IMTG accumulation via increased DGAT1 expression. This change came with an increase in the expression of oxidative metabolism factors in the skeletal muscle and did not induce insulin resistance. Collectively, these results suggest that olive oil would be a new exercise-mimetic food; however further studies are required to clarify the detailed mechanism underlying the effects of olive oil on skeletal muscle.

## Figures and Tables

**Table 1:** The composition of experimental diets.

Ingredient (g/kg)	Control	Olive Oil
Casein	200	200
l-Cystine	3	3
Corn Starch	397.486	397.486
Maltodextrin 10	132	132
Sucrose	100	100
Cellulose, BW200	50	50
Soybean oil	70	-
Olive oil	-	70
Mineral Mix S10022G	35	35
Vitamin Mix V10037	10	10
Choline Bitartrate	2.5	2.5
t-Butylhydroquinone	0.028	0.028
FD&C Yellow Dye, #5	0.05	-
FD&C Red Dye, #40	-	0.05

**Table 2:** The fatty acid composition of the dietary fats in experimental diets (%).

Fatty Acid	Soybean Oil	Olive Oil
C16:0 (Palmitic acid)	10.4	11.5
C16:1 (Palmitoleic acid)	-	1.2
C18:0 (Stearic acid)	3.8	2.3
C18:1 (Oleic acid)	24.3	70.5
C18:2 (Linoleic acid)	53.5	13.0
C18:3 (Linolenic acid)	7.8	0.6
C20:0 (Arachidic acid)	-	0.4
C20:1 (Eicosenoic acid)	-	0.2
Others	0.2	0.3
SFA	14.2	14.2
MUFA	24.3	71.9
PUFA	61.3	13.6

SFA: saturated fatty acid, MUFA: monounsaturated fatty acid, PUFA: polyunsaturated fatty acid.

**Table 3:** List of primer sequences for reverse transcription-quantitative PCR.

Gene	Primer Sequences (5′-3′)	Gene	Primer Sequences (5′-3′)
SREBP1	F: GGTTTTGAACGACATCGAAGA	HSL	F: GCGCTGGAGGAGTGTTTTT
	R: CGGGAAGTCACTGTCTTGGT		R: CGCTCTCCAGTTGAACCAAG
PPARγ	F: GAAAGACAACGGACAAATCACC	LPL	F: TTTGTGAAATGCCATGACAAG
	R: GGGGGTGATATGTTTGAACTTG		R: CAGATGCTTTCTTCTCTTGTTTGT
PPARδ	F: CTCACCGAGTTCGCCAAG	ATGL	F: TGACCATCTGCCTTCCAGA
	R: AGGGTCACCTGGTCATTGAG		R: TGTAGGTGGCGCAAGACA
PPARα	F: TTCCAAAGCAAGGTCTGAGG	CPT1β	F: GTCATGGCACTGGGTATGGT
	R: GGATGGCACCAAGGACAGTA		R: GGGATGCGTGTAGTGTTGAA
ACSL1	F: AAAGATGGCTGGTTACACACG	PDK4	F: CGCTTAGTGAACACTCCTTCG
	R: CGATAATCTTCAAGGTGCCATT		R: CTTCTGGGCTCTTCTCATGG
DGAT1	F: GTCAAGGCCAAAGCTGTCTC	GCK	F: TCCCTGTAAGGCACGAAGAC
	R: AACACAAAGTAGGAGCAAAGATGA		R: ACGATGTTGTTCCCTTCTGC
DGAT2	F: GGCGCTACTTCCGAGACTAC	GLUT4	F: GATTCTGCTGCCCTTCTGTC
	R: TGGTCAGCAGGTTGTGTGTC		R: CGGTCAGGCGCTTTAGAC
CD36	F: TGGAGCTGTTATTGGTGCAGT	MPC1	F: TGAATAGCCGAGAGTCCCTAAA
	R: GGTTCCTTCTTCAAGGACAACTT		R: TGATGAAGACAAATAAGGTTTAGCA
FASN	F: GCTGCTGTTGGAAGTCAGC	UCP3	F: TACCCAACCTTGGCTAGACG
	R: AGTGTTCGTTCCTCGGAGTG		R: GTCCGAGGAGAGAGCTTGC
ACACA	F: GCGTCGGGTAGATCCAGTT	PGC1α	F: TGAAAGGGCCAAACAGAGAG
	R: CTCAGTGGGGCTTAGCTCTG		R: GTAAATCACACGGCGCTCTT
PLIN5	F: GTCGGAGAAGCTGGTGGAC	MyHC1	F: GAGCAGCAGGTGGATGATCT
	R: TCAGCTGCCAGGACTGCTA		R: GCTTGGCTCGCTCTAGGTC
TBP	F: GGGGAGCTGTGATGTGAAGT	MyHC2A	F: AAAGCTCCAAGGACCCTCTT
	R: CCAGGAAATAATTCTGGCTCAT		R: AGCTCATGACTGCTGAACTCAC

SREBP1, sterol regulatory element binding protein 1; PPARγ, δ, α, peroxisome proliferator activated receptor gamma, delta, alpha; ACSL1, acyl-CoA synthetase long-chain family member 1; DGAT1,2, diacylglycerol O-acyltransferase1, 2; FASN, fatty acid synthase; ACACA, acetyl-CoA carboxylase alpha; PLIN5, perilipin 5; HSL, hormone-sensitive lipase; LPL, lipoprotein lipase; ATGL, adipose triglyceride lipase; CPT1β, carnitine palmitoyltransferase 1β; PDK4, pyruvate dehydrogenase kinase 4; GCK, glucokinase; GLUT4, glucose transporter 4; MPC1, mitochondrial pyruvate carrier 1; UCP3, uncoupling protein 3; PGC1α, PPARγ coactivator 1 alpha; MyHC1, 2A, myosin heavy chain 1, 2A; TBP, TATA-box-binding protein.

**Table 4:** Growth performance, tissue weights, and serum biochemical components.

	Control			Olive Oil		
Body weight gain and food intake						
Final body weight (g)	25.8	±	0.18	25.1	±	0.72
Body weight gain (g)	3.88	±	0.36	3.50	±	0.51
Total food intake (g)	149.1	±	2.3	144.4	±	0.3
Tissue weight (mg)						
Soleus muscle	12.0	±	0.4	11.2	±	0.3
EDL muscle	12.1	±	0.4	11.9	±	0.3
Gastrocnemius muscle	276.6	±	9.2	271.4	±	6.4
Epididymal fat	479.7	±	47.3	496.9	±	44.2
Perirenal fat	193.9	±	11.5	154.1	±	20.1
Inguinal fat	381.7	±	32.0	349.5	±	34.2
Brown adipose tissue	175.4	±	17.2	161.9	±	13.0
Liver	1109.8	±	23.9	1073.0	±	45.1
Serum biochemical component						
Glucose (mg/100 mL)	206.4	±	17.4	212.3	±	4.7
TAG (mg/100 mL)	102.1	±	17.8	115.8	±	19.6
NEFA (mEq/L)	0.87	±	0.20	0.99	±	0.14

Values are means ± SE for 8 mice. EDL, extensor digitorum longus; TAG, triacylglycerol; NEFA, non-esterified fatty acid.

## References

[1] Schiaffino S., Reggiani C. (2011). Fiber types in Mammalian skeletal muscles. Physiol. Rev..

[2] Van Loon L.J.C. (2004). Use of intramuscular triacylglycerol as a substrate source during exercise in humans. J. Appl. Physiol..

[3] Blaauw B., Schiaffino S., Reggiani C. (2013). Mechanisms modulating skeletal muscle phenotype. Compr. Physiol..

[4] Hawley J.A., Hargreaves M., Joyner M.J., Zierath J.R. (2014). Integrative biology of exercise. Cell.

[5] Goodpaster B.H., He J., Watkins S., Kelley D.E. (2001). Skeletal muscle lipid content and insulin resistance: Evidence for a paradox in endurance-trained athletes. J. Clin. Endocrinol. Metab..

[6] Shepherd S.O., Cocks M., Tipton K.D., Ranasinghe A.M., Barker T.A., Burniston J.G., Wagenmakers A.J.M., Shaw C.S. (2013). Sprint interval and traditional endurance training increase net intramuscular triglyceride breakdown and expression of perilipin 2 and 5. J. Physiol..

[7] Ahamad J., Toufeeq I., Khan M.A., Ameen M.S.M., Anwer E.T., Uthirapathy S., Mir S.R., Ahmad J. (2019). Oleuropein: A natural antioxidant molecule in the treatment of metabolic syndrome. Phytother. Res..

[8] Rodríguez V.M., Portillo M.P., Picó C., Teresa Macarulla M., Palou A. (2002). Olive oil feeding up-regulates uncoupling protein genes in rat brown adipose tissue and skeletal muscle. Am. J. Clin. Nutr..

[9] Mizunoya W., Iwamoto Y., Shirouchi B., Sato M., Komiya Y., Razin F.R., Tatsumi R., Sato Y., Nakamura M., Ikeuchi Y. (2013). Dietary fat influences the expression of contractile and metabolic genes in rat skeletal muscle. PLoS ONE.

[10] Folch J., Lees M., Sloane Stanley G. (1957). A Simple method for the isolation and purification of total lipides from animal tissues. J. Biol. Chem..

[11] Komiya Y., Sawano S., Mashima D., Ichitsubo R., Nakamura M., Tatsumi R., Ikeuchi Y., Mizunoya W. (2017). Mouse soleus (slow) muscle shows greater intramyocellular lipid droplet accumulation than EDL (fast) muscle: Fiber type-specific analysis. J. Muscle Res. Cell Motil..

[12] Komiya Y., Mizunoya W., Kajiwara K., Yokoyama I., Ogasawara H., Arihara K. (2020). Correlation between skeletal muscle fiber type and responses of a taste sensing system in various beef samples. Anim. Sci. J..

[13] Goodpaster B.H. (2020). CrossTalk proposal: Intramuscular lipid accumulation causes insulin resistance. J. Physiol..

[14] Kim Y.J., Greimel P., Hirabayashi Y. (2018). GPRC5B-Mediated Sphingomyelin Synthase 2 Phosphorylation Plays a Critical Role in Insulin Resistance. iScience.

[15] Sawano S., Komiya Y., Ichitsubo R., Ohkawa Y., Nakamura M., Tatsumi R., Ikeuchi Y., Mizunoya W. (2016). A one-step immunostaining method to visualize rodent muscle fiber type within a single specimen. PLoS ONE.

[16] Cree M.G., Paddon-Jones D., Newcomer B.R., Ronsen O., Aarsland A., Wolfe R.R., Ferrando A. (2010). Twenty-eight-day bed rest with hypercortisolemia induces peripheral insulin resistance and increases intramuscular triglycerides. Metabolism.

[17] Badin P.M., Vila I.K., Louche K., Mairal A., Marques M.A., Bourlier V., Tavernier G., Langin D., Moro C. (2013). High-fat diet-mediated lipotoxicity and insulin resistance is related to impaired lipase expression in mouse skeletal muscle. Endocrinology.

[18] Murase T., Haramizu S., Shimotoyodome A., Tokimitsu I., Hase T. (2006). Green tea extract improves running endurance in mice by stimulating lipid utilization during exercise. Am. J. Physiol. Regul. Integr. Comp. Physiol..

[19] Fan W., Waizenegger W., Lin C.S., Sorrentino V., He M.X., Wall C.E., Li H., Liddle C., Yu R.T., Atkins A.R. (2017). PPARδ Promotes Running Endurance by Preserving Glucose. Cell Metab..

[20] Sharman I.M. (1973). Nutrition and Athletic Performance. Nutr. Bull..

[21] Ishihara K., Oyaizu S., Onuki K., Lim K., Fushiki T. (2000). Chronic (-)-hydroxycitrate administration spares carbohydrate utilization and promotes lipid oxidation during exercise in mice. J. Nutr..

[22] Kiens B. (2006). Skeletal muscle lipid metabolism in exercise and insulin resistance. Physiol. Rev..

[23] Horton J.D., Goldstein J.L., Brown M.S. (2002). SREBPs: Activators of the complete program of cholesterol and fatty acid synthesis in the liver. J. Clin. Investig..

[24] Yu J., Li Y., Zou F., Xu S., Liu P. (2015). Phosphorylation and function of DGAT1 in skeletal muscle cells. Biophys. Rep..

[25] Liu L., Shi X., Cheol S.C., Shulman G.I., Klaus K., Nair K.S., Schwartz G.J., Zhang Y., Goldberg I.J., Yu Y.H. (2009). Paradoxical coupling of triglyceride synthesis and fatty acid oxidation in skeletal muscle overexpressing DGAT1. Diabetes.

[26] Li T., Xu D., Zuo B., Lei M., Xiong Y., Chen H., Zhou Y., Wu X. (2013). Ectopic overexpression of porcine DGAT1 increases intramuscular fat content in mouse skeletal muscle. Transgenic Res..

[27] Yang F., Wei Z., Ding X., Liu X., Ge X., Song G., Li G., Guo H. (2013). Upregulation of triglyceride synthesis in skeletal muscle overexpressing DGAT1. Lipids Health Dis..

[28] Chantranupong L., Scaria S.M., Saxton R.A., Gygi M.P., Shen K., Wyant G.A., Wang T., Harper J.W., Gygi S.P., Sabatini D.M. (2016). The CASTOR Proteins Are Arginine Sensors for the mTORC1 Pathway. Cell.

[29] Fujiwara Y., Tsukahara C., Ikeda N., Sone Y., Ishikawa T., Ichi I., Koike T., Aoki Y. (2017). Oleuropein improves insulin resistance in skeletal muscle by promoting the translocation of GLUT4. J. Clin. Biochem. Nutr..

[30] Hadrich F., Garcia M., Maalej A., Moldes M., Isoda H., Feve B., Sayadi S. (2016). Oleuropein activated AMPK and induced insulin sensitivity in C2C12 muscle cells. Life Sci..

[31] Watanabe N., Komiya Y., Sato Y., Watanabe Y., Suzuki T., Arihara K. (2020). Oleic acid up-regulates myosin heavy chain (MyHC) 1 expression and increases mitochondrial mass and maximum respiration in C2C12 myoblasts. Biochem. Biophys. Res. Commun..

[32] Tierney A.C., Roche H.M. (2007). The potential role of olive oil-derived MUFA in insulin sensitivity. Mol. Nutr. Food Res..

[33] Marika M., Egeria S., Maria Annunziata C., Giuseppe S., Tiziano V., Raffaele D.C., Nadia C. (2020). Effects of Olive Oil on Blood Pressure: Epidemiological, clinical, and mechanistic evidence. Nutrients.

